# Experiences of adults who underwent peritoneal dialysis in childhood: retrospective and qualitative study

**DOI:** 10.1007/s00467-026-07410-7

**Published:** 2026-06-25

**Authors:** Ayşen Toktay, Kader Mert

**Affiliations:** 1 Health Sciences University Behçet Uz Child’s Diseases and Surgery Training and Research Hospital, Izmir, Turkey; 2 Department of Nursing, Faculty of Health Sciences, Public Health Nursing, Bakırçay University, Izmir, Turkey

**Keywords:** Adulthood, Children, Peritoneal dialysis, Experience, Semi-structured interview

## Abstract

**Background:**

Although there are numerous studies on the parents of children undergoing peritoneal dialysis (PD) treatment, there are only a limited number of studies examining what children undergoing PD treatment experience until adulthood and their experiences with PD treatment during childhood. The aim of this study is to examine the changes that occur in the lives of adults who received PD treatment during childhood after diagnosis and their experiences related to the treatment process.

**Methods:**

This retrospective and qualitative study was conducted with 18 adult participants living in the province of Izmir in Turkey who had received at least 6 months of PD treatment during childhood. Participants were selected using snowball sampling. A semi-structured interview form was used as the data collection tool. Study data were collected through face-to-face interviews using the individual in-depth interview technique. Data obtained from interviews were analyzed using Braun and Clarke’s thematic analysis approach.

**Results:**

The study identified four main themes: psychological effects, daily life effects, social effects, and academic effects. Psychological effects include the sub-themes of fear, shock, sadness, hopelessness, and shame. Daily life effects include the sub-themes of changes in eating habits, changes in body image, difficulty taking medication, repeated hospitalizations, stress coping, and adaptation. Social effects include social isolation, peritoneal dialysis room, social support, and personal life. Academic effects include the sub-themes of absenteeism, taking a break from education, academic failure, and being forced to change career choice.

**Conclusion:**

The findings from this research will provide guidance for a deeper understanding of the needs of children undergoing PD today and for initiatives planned in this context.

**Graphical abstract:**

**Figure:**
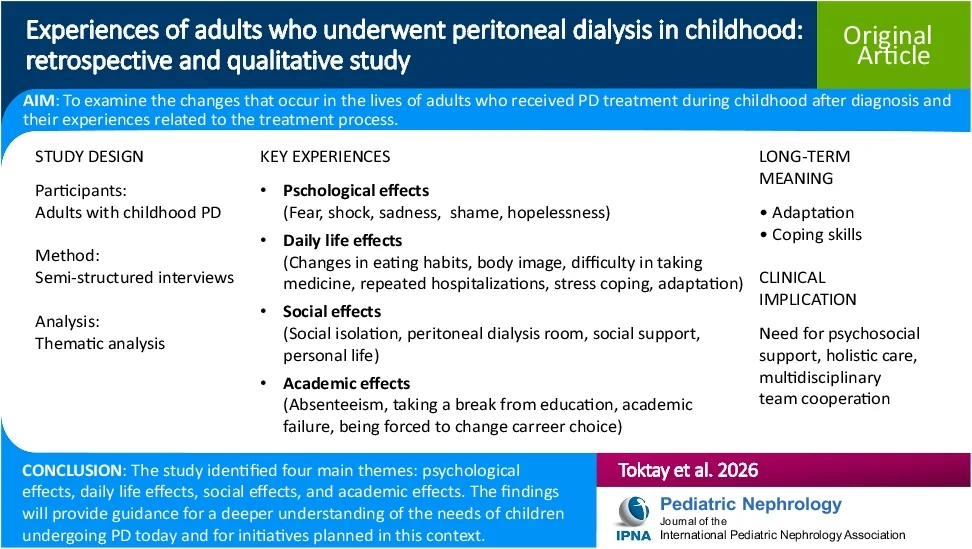
A higher resolution version of the Graphical abstract is available as Supplementary information

**Supplementary Information:**

The online version contains supplementary material available at https://doi.org/10.1007/s00467-026-07410-7.

## Introduction

Only approximately 10–15% of dialysis patients are treated with peritoneal dialysis (PD) [1, 2]. In Türkiye, a total of 94,461 patients require kidney replacement therapy, and these patients are treated with either dialysis or kidney transplantation. In Türkiye, approximately 67.4% of patients receiving kidney replacement therapy are treated with hemodialysis, 4.1% with PD, and 1.4% with home hemodialysis. On average, 23,520 patients undergo kidney transplantation annually. In the presence of a suitable donor, kidney transplantation is the preferred treatment modality for stage 5 chronic kidney disease (CKD 5) in children compared to dialysis and is associated with better growth, development, and quality of life outcomes [3]. PD is more frequently preferred than hemodialysis in the treatment of CKD 5 in children; the main reasons for this preference include its applicability at home, the lack of need for vascular access, and its provision of greater hemodynamic stability [1, 2]. As of 2024, there are a total of 405 pediatric patients on PD aged 0–19 years in Türkiye [4]. Data on pediatric PD patients in Izmir are not definitively known. PD often serves as a bridge to kidney transplantation [5]. PD can be performed manually as Continuous Ambulatory Peritoneal Dialysis (CAPD) or as Automated Peritoneal Dialysis (APD) using automated cyclers overnight [6]. Due to the nocturnal nature of APD and the high responsibility of the caregiver during the daily treatment process, family members, especially mothers, assume significant responsibilities [7]. The motivation of caregivers plays a critical role in the sustainability of PD [8]. However, unlike adult PD practices, pediatric PD involves a family-centered care approach, the active involvement of caregivers, and specific needs related to growth and development, as well as the maintenance of education and social life [7]. These characteristics indicate that children undergoing PD experience a care process that is not only medical but also psychosocially distinct and more complex.

Individuals undergoing PD are reported to experience various physical, psychological, and social difficulties in their daily lives. Existing qualitative studies have predominantly focused on the immediate experiences of children receiving PD treatment and their families, revealing themes such as fear, uncertainty, treatment burden, and dependence on caregivers. However, there is limited evidence regarding how individuals who received PD treatment during childhood interpret these experiences in later life and the long-term effects of this process.

The limited number of qualitative studies conducted with children undergoing PD and their caregivers indicates that experiences such as fear, uncertainty, anxiety related to medical procedures and devices, dependence on caregivers, and restrictions in daily life are prominent during the treatment process [9, 10]. While these studies are valuable in revealing experiences specific to the treatment environment and process, they mostly reflect short-term perceptions shaped by the ongoing treatment process. In contrast, there is limited evidence regarding how individuals who received PD during childhood evaluate these experiences years later and the lifelong effects of this process.

Therefore, this study aims to retrospectively examine the experiences of adults who underwent PD during childhood, to reveal the long-term psychosocial, social, and educational effects of early life experiences, and to provide a deeper and more comprehensive perspective to the existing literature.

## Methods

### Study design and participants

This study was a retrospective qualitative research designed to understand the life experiences of individuals who underwent PD treatment during childhood. Qualitative research is important for understanding individuals’ worlds of meaning, emotions, and experiences within a social context [11]. The study population consisted of adults living in the province of Izmir, Turkey, who underwent PD treatment during childhood. Snowball sampling was used in the study. Snowball sampling is a non-probability sampling technique based on referral and progressing through participants recommending each other, used especially in situations where it is difficult to reach individuals with a specific characteristic [12]. After the initial participants were identified according to the inclusion criteria, they were asked to share information about the study with other individuals who had received PD during childhood and might be eligible to participate. Due to confidentiality, the contact information of potential participants was not shared directly; instead, individuals who were willing to participate contacted the researchers themselves. The sampling process continued until data saturation was achieved. In this study, due to the rarity of individuals who underwent PD during childhood in society, this sampling method was employed. During the process of sample selection, the principle of data saturation was adhered to, and when similar statements began to be reiterated, the recruitment of new participants was terminated. In order to achieve this objective, individual semi-structured in-depth interviews were conducted with a total of 18 participants.

### Semi-structured individual in-depth interview form

This form consists of five open-ended questions aimed at gaining an in-depth understanding of experiences with PD during childhood.

- How did you react when you learned you would be receiving peritoneal dialysis treatment as a child? How did you feel?
- What changes occurred in your life during the peritoneal dialysis treatment process? How did these changes affect you?
- What are your most memorable experiences from the peritoneal dialysis treatment period? Can you share any unforgettable memories?

- How did you cope with peritoneal dialysis treatment as a child?
- What were the implications of the peritoneal dialysis treatment process into adulthood?

During the data collection process, opinions were sought from eight experts to ensure the content validity of the interview form, and the feasibility of the interview form was tested through a pilot study. Before initiating the data collection process, a pilot study was conducted to evaluate the clarity and feasibility of the interview guide. Within the scope of the pilot study, semi-structured interviews were conducted with two participants who met the inclusion criteria and represented the target population. During these interviews, the clarity, sequence, and comprehensibility of the questions were assessed. As a result of the pilot application, minor revisions were made to the wording of some questions, and the flow of the interview was improved. This method is one of the standard practices recommended to increase reliability and validity in qualitative research [13].

### Data collection

Research data was collected between March 30 and June 30, 2025. Prior to the interviews, the date and time were scheduled with the participants, and the interviews were conducted face-to-face in the participants' homes using an individual in-depth form to ensure their privacy and comfort. Both verbal and written consent was obtained from the participants during the interviews. With the participants' permission, audio recording devices were used during the data collection process. To ensure consistency in the data collection process, all interviews and data collection procedures were conducted by a single researcher experienced in qualitative research. The interviews lasted between 18 and 37 min on average, and the audio recordings were transcribed word for word. The data collection process was continued until data saturation was achieved. The data collection process was continued until the point at which the data obtained from the interviews began to repeat, no new codes, themes, or subthemes emerged, and the existing themes were supported by sufficiently rich data. The data collection process was terminated when the data obtained from new participants largely overlapped with the information obtained previously and did not create new themes or categories.

### Ethical considerations

Before starting the research, the necessary legal permits and ethical committee approval were obtained from the Ethics Committee of the Izmir Bakırcay University (Ethics Committee Decision No: 2167/Date: 03/29/2025). Participants were thoroughly informed about the objectives and procedures of the study, and both written and verbal informed consent were obtained. All procedures followed the principles of the Declaration of Helsinki [14].

### Data analysis

The data obtained in this study were evaluated using the qualitative content analysis method. Audio recordings obtained from the interviews were transcribed verbatim, and each interview text was read by the researchers. The data were analyzed in accordance with the thematic analysis framework proposed by Braun and Clarke [15], with a view to ensuring a consistent and systematic approach throughout the analytic process. The coding of the data was carried out manually by the researchers without the use of any qualitative data analysis software. The processes of coding and theme development were conducted systematically, and consensus was achieved among the researchers throughout the analysis. The analysis was conducted in six sequential steps (Table 1). In the initial phase, the transcripts were meticulously reviewed to establish familiarity with their content and meaning. In the subsequent phase, codes were allocated to particular segments of the data and meticulously scrutinized to ascertain their pertinence to the research questions and overarching objectives. The data was independently coded by both authors (blind coding). In the third step of the process, the identified codes were grouped together to formulate broader preliminary themes. In the fourth step of the process, these themes were reviewed, and the supporting data for each was validated. The fifth step entailed a meticulous re-reading of the transcripts to ensure that no significant data had been overlooked. The initial themes and sub-themes were then subjected to a process of refinement and systematic organization. In the sixth step of the research process, the findings were synthesized into a comprehensive narrative that encapsulated the study’s core themes. This narrative was further substantiated by direct quotations from the participants, thereby enhancing the credibility and authenticity of the study’s findings.

**Table 1:** Six-phase thematic analysis approach by Braun and Clarke (2006)

Phase	Description
1. Familiarization with the data	Interview recordings were transcribed verbatim, and the transcripts were read repeatedly by the researchers to achieve familiarity with the data
2. Generating initial codes	Meaningful segments of the data were systematically coded, and data relevant to the research questions were identified
3. Searching for themes	Similar codes were grouped together to develop potential themes and subthemes
4. Reviewing themes	The identified themes were compared with the dataset to evaluate their consistency, and necessary revisions were made
5. Defining and naming themes	Each theme and subtheme was clearly defined, their scope was determined, and they were meaningfully named
6. Reporting the findings	The results of the analysis were systematically reported and supported with participants’ quotations

## Results

Participants’ ages ranged from 18 to 37. The majority of participants were women and single individuals. When examining the treatment process data, a large proportion of participants started with PD, some individuals underwent hemodialysis, and most continued treatment with kidney transplantation (Table 2). 66.6% (*n*: 12) of the participants were female. The average age at the start of PD treatment was (12.11 ± 2.51 years) (min: 9–max: 17). 55.5% (*n*: 10) of participants received PD via ADP, and 44.5% (*n*: 8) via CAPD. The average duration of PD was (4.66 ± 4.43 years) (min: 8 months–max: 19 years) (Table 3). The themes and sub-themes created as a result of the research data are shown in Table 4.

**Table 2:** Participants’ characteristics according to age, gender, marital status, and treatment processes

Participants (P)	Age	Gender	Marital status	Peritoneal dialysis	Treatment process
P1	29	Female	Single	APD	APD (13–17 age, 4 years), KT (17 age and later, 12 years)
P2	33	Female	Single	APD	APD (11–13 age, 2 years), hemodialysis (32–33 age, 1 year), KT (14–32 age, 17 years)
P3	32	Male	Single	CAPD	CAPD (11–30 age, 19 years), hemodialysis (30–32 age, 2 years)
P4	30	Male	Single	APD	APD (10–21 yaş, 11 years), hemodialysis (28–32 age, 2 years), KT (21–28 age, 7 years)
P5	28	Male	Single	APD	APD (16–18.5 age, 2.5 years), KT (18.5–28 age, 10 years)
P6	22	Female	Single	APD	APD (14–16 age, 2 years), KT (16–22 age, 6 years)
P7	34	Female	Single	CAPD	CAPD (16–21 age, 5 years), KT (22–34 age, 12 years)
P8	19	Female	Single	APD	ADP (9 age, 11 ay), KT (10–19 age, 9 years)
P9	18	Female	Single	APD	APD (12–14 age, 2 years), KT (14–18 age, 4 years)
P10	27	Male	Single	CAPD	CAPD (10–14age, 4 years), hemodialysis (26–27 age, 1 year), KT (14–26 age, 12 years)
P11	28	Male	Single	CAPD	CAPD (12–15 age, 2 years), KT (16–28 age, 12 years)
P12	21	Female	Single	CAPD	CAPD (10–17 age, 7 yıl), KT (18–21 age, 3 yıl)
P13	33	Female	Single	CAPD	CAPD (11–16 age, 5 years), hemodialysis (17–24 age, 7 years), KT (24–31 age, 6 years)
P14	22	Female	Single	APD	APD (10–13 age, 3 years), hemodialysis (13 age, 2 months), KT (14–22 age, 8 years)
P15	26	Female	Married	APD	APD (12–15 age, 3 years), KT (16–26 age, 10 years)
P16	31	Male	Married	CAPD	CAPD (9–17 age, 8 years), KT (18–31 age, 13 years)
P17	19	Female	Single	APD	APD (15–17 age, 2 years), hemodialysis (17, 6 months), KT (18–19 age, 1 year)
P18	37	Female	Single	CAPD	CAPD (17–18 age, 8 months), KT (19–37 age, 18 years)

*APD*, Automated Peritoneal Dialysis; *CAPD*, Continuous Ambulatory Peritoneal Dialysis; *KT*, kidney transplantation

**Table 3:** Participants’ childhood experiences with peritoneal dialysis

Characteristic	*n* (%)
Gender	
Female	12 (66.6)
Male	6 (33.4)
Type of peritoneal dialysis	
Automated Peritoneal Dialysis (ADP)	10 (55.5)
Continuous Ambulatory Peritoneal Dialysis (CAPD)	8 (44.5)
Age at initiation of peritoneal dialysis	
Age (years) (mean ± SD)	(12.11 ± 2.51 years)
(Min–max)	(Min: 9 age–max: 17 age)
Duration of peritoneal dialysis treatment	
Years (mean ± SD)	(4.66 ± 4.43 years)
(Min–max)	(Min: 8 months–max: 19 years)
Total	18 (100)

**Table 4:** Themes and sub-themes regarding the effects of peritoneal dialysis in childhood

Themes	Sub-themes	Areas of concern specified by interviews
Psychological effects	Fear	Dressing Peritoneal dialysis machine Large water bags Peritoneal catheter
	Shock	
	Sadness	Mothers’ sadness Pity
	Hopelessness	
	Shame	Peritoneal catheter
Daily life effects	Changes in eating habits	Salt-free diet Water restriction Not being able to eat junk food (chocolate, chips) and to drink carbonated beverages
	Changes in body image	Peritoneal catheter Growth retardation Changes in clothing selection
	Difficulty in taking medicine	Use of multiple medications
	Repeated hospitalizations	Infection High phosphorus levels
	Stress coping	Watching movies Reading books Dancing Doing makeup Writing down feelings Listening to music Social media Daydreaming Emotional eating behavior
	Adaptation	Taking responsibility for treatment
Social effects	Social isolation	Withdrawal Difficulty participating in social activities Travel restrictions Restrictions on swimming in the sea Inability to play favorite games Being excluded in games Refusing to give up favorite games
	Peritoneal dialysis room	Toilet Television Internet Wanting the electricity to be turned off Disinfectant smell
	Social support	Support from children undergoing peritoneal dialysis Family support Support from close friends
	Personal life	Rejection
Academic effects	Absenteeism	Leaving class early Not wanting to go to school
	Taking a break from education	
	Academic failure	
	Being forced to change career choice	

### Psychological effects

Most participants reported experiencing intense fear, shock, sadness, hopelessness, and shame from the very beginning of the diagnosis and treatment process.

#### Fear

All participants experienced fear. Participants stated that the source of their fear was dressing changes, the PD machine, large PD water bags, and catheters.

The dressing scared me more than dialysis; I fainted while the dressing was being applied (P7)

When I first heard the sound of the machine, my heart felt like it was going to jump out of my chest (P12)

The large water bags became my nightmares... sometimes I would wake up with a start from my sleep (P16)

#### Shock

Most participants described the emotions they experienced upon receiving their diagnosis as “life suddenly being turned upside down.”

One day I was at school, the next day I was on the operating table. I was in shock. Everything had changed before I knew what was happening. (P2)

Some participants stated that the shock they experienced was not only the result of encountering a physical illness, but also of being separated from their childhood routines.

Suddenly, I was separated from all my friends, school, and home. I felt as if my life had been taken away from me. (P7)

#### Sadness

In the sadness sub-theme, most participants expressed that they were affected not only by their own emotional responses but also by the sadness experienced by family members, especially their mothers.

**Mothers’ sadness**

I was sad, but my mother was even sadder. Seeing her like that affected me even more. (P8)

**Pity**

Some participants mentioned that well-intentioned but overly sensitive approaches eventually turned into a feeling of pity, which was very upsetting.

“Everyone looked at me with pity… I felt even worse.” (P9).

#### Hopelessness

Most participants stated that they experienced intense feelings of hopelessness at times during their illness and treatment.

Sometimes I thought it would never end. Being hooked up to machines was very difficult. I didn’t know when I would return to normal, and I felt hopeless in that situation. (P8)

I said there was no escape after that. (P11)

#### Shame

Most participants stated that they experienced intense feelings of shame during the treatment process due to the catheter.

I would think about what people would think if they saw my catheter, and I would feel very ashamed. (P7)

During breaks at school, while everyone else was playing, I would look in the mirror to see if my catheter was visible (P11)

### Daily life activities

#### Changes in eating habits

Dietary restrictions during PD, particularly salt-free eating, limited water consumption, and avoiding junk food and acidic beverages, created significant difficulties during childhood.

**Salt-free diet**

Our biggest crisis was the salt problem, I know I often left the table hungry... especially that pickle crisis at the table. (P7)

**Water restriction**

For some participants, water restriction was particularly challenging for children, especially during the summer months.

Other than that, water was forbidden. I don’t know, we were sweating in the summer, we were running around. The children were drinking water right in front of me. (P10)

**Not being able to eat junk food (chocolate, chips) and to drink carbonated beverages**

Some participants stated that they found it difficult to comply with the restrictions on junk food and fizzy drinks.

When I was outside with my friends, whatever junk food they ate, I ate too. I didn’t really care, I didn’t hide it either. (P14)

#### Changes in body image

**Peritoneal catheter**

The presence of a peritoneal catheter in particular created difficulties for most participants in terms of both physical comfort and social visibility.

The catheter was attached….it wasn’t comfortable, you couldn’t sleep, you couldn’t take a shower. (P10)

**Growth retardation**

Some participants experienced problems with their body image, particularly due to growth retardation.

I was smaller than my peers in height and weight. (P8)

I was very troubled by my short stature …There were no clothes for me in stores. (P15)

**Changes in clothing selection**

Most participants mentioned experiencing difficulties in choosing clothes.

I just couldn’t wear tight pants (P10)

#### Difficulty in taking medication

**Use of multiple medications**

The majority of participants highlighted difficulty in taking medication as one of the most common challenges they faced.

We had so many medications. (P13)

Because I had never used medication before, it was naturally difficult to suddenly use so many. (P9)

#### Repeated hospitalizations

**Infection**

Some participants experienced complications such as peritonitis, which prolonged their hospital stays and caused them to develop negative feelings toward treatment.

I also had peritonitis a couple of times, and those times were really bad. (P9)

**High phosphorus levels**

Some participants stated that the difficulty of controlling their diet during childhood led to occasional increases in phosphorus levels, resulting in hospitalizations.

I loved chocolate and dairy products, but the doctor forbade them. When I couldn’t resist and ate them, my phosphorus levels would immediately rise in the tests (P4)

I’m afraid of high phosphorus levels in the tests. I still check my blood tests directly for phosphorus (P15)

#### Stress coping

Most participants preferred listening to music, watching movies, reading books, dancing, spending time on social media accounts, writing down their feelings, doing makeup, and daydreaming as ways to cope with stress.

When I put on my headphones and listened to music, the world would stop, and it would just be me and the songs (P6)

At night, I would write down the things I couldn’t tell anyone in my notebook. (P11)

I would imagine the day I would get better; if I didn’t have my dreams, I wouldn’t be this strong (P7)

Some participants stated that they struggled to cope when stressed and ate emotionally.

The more upset I get, the more I eat (P17)

#### Adaptation

Some participants took photos with their catheters, decorated them, or freely displayed them, which is considered part of the acceptance process. One participant expressed their adaptation to treatment by saying they gave the dialysis machine a name.

My machine was very different for me. It had its own name, Mr. Iron. (P17)

**Taking responsibility for treatment**

Over time, participants have become more independent by taking on a more active role in the dialysis and care process.

…It was as if I were a nurse, telling my mother, ‘You shouldn’t do it this way, you should do it that way; you don’t know how, but I can do it’… (P 7)

### Social effects

#### Social isolation

**Withdrawal**

Most participants stated that they developed an increasingly introverted personality during the course of their illness and withdrew from social interactions.

The illness really closed me off. (P11)

Even after the illness ended, I couldn’t be as social as I used to be. (P18)

**Difficulty participating in social activities**

Most participants indicated that they experienced restrictions in their social relationships.

I never went out … I went out wearing a mask, I didn’t like it, so my relationships were interrupted. (P11)

**Travel restrictions**

Most participants stated that peritoneal dialysis machines limited their freedom to travel and go on trips.

My family would go outside the city. I couldn’t go because of dialysis (P13)

**Restrictions on swimming in the sea**

Some participants stated that they had difficulty swimming in the sea and participating in daily social activities.

as I moved, I felt bloating in my stomach... I couldn’t move much. I couldn’t go outside anyway (P14)

**Inability to play favorite game**

Some participants stated that they could not play their favorite games.

I was afraid to play…what if the ball hit my stomach…would something happen to my catheter. (P13)

**Being excluded from games**

Some participants stated that they experienced exclusion in games.

no one chased me in the game… the kids didn’t throw the ball to me (P7)

**Refusing to give up favorite games**

However, some participants also stated that they did not give up the games they loved despite all the restrictions.

Blood was coming from the catheter, but I was playing ball. What did I say to myself. (P16)

**Peritoneal dialysis room**

Most participants described the peritoneal dialysis room at home as a separate living space.

I was lying alone in the room, only the sound of the machines beside me. (P8)

**Wanting the electricity to be turned off**

One participant described the temporary sense of freedom that power outages at home gave them:

I was so happy when the power went out. I was free... My mom was calling about the power outage... I told her not to rush, that it would come back... When I was a child, I didn’t have to worry about dialysis like this. (P7)

**Disinfection smell**

Participants also stated that they still remember the smell of disinfectant in the room.

My room was like a hospital room… I still smell bleach when I enter that room. (P10)

#### Social support

**Support from children receiving peritoneal dialysis**

All participants received support from friends undergoing PD treatment.

M…was there. L… came later… Our conversations were nice, we always talked about peritoneal dialysis (P8)

Meeting children like me at the hospital made me feel better. Talking to them, knowing we were going through the same thing, made me feel better. (P4)

**Family support**

The majority of participants emphasized that they received support from their families.

My family took great care of me, as if I were a glass vase. (P4)

**Support from close friends**

Some participants emphasized that their friends empathized with them and sometimes made sacrifices.

My friends didn’t even go to concerts for me, just because I couldn’t go, they didn’t go either. (P5)

#### Personal life

**Rejection**

Most participants emphasized that the illness and treatment process also caused obstacles in emotional relationships.

My boyfriend’s family didn’t want me (P7)

### Academic life

#### Absenteeism

**Leaving classes early**

Most participants stated that they frequently experienced interruptions in their education due to peritoneal dialysis treatment.

I would leave class early because I had to go for dialysis. (P7)

**Not wanting to go to school**

Some participants did not want to go to school during their treatment.

When I went to school, we would end up being hospitalized 3–4 weeks later anyway… then I didn’t want to go to school. (P12)

## Taking a break from education

Some participants stated that they took a break from their education, while others stated that they dropped out completely.

I continued at an open high school. I dropped out a year later. (P11)

I took a two-year break from school. (P15)

## Academic failure

Most participants stated that they experienced academic failure due to absenteeism from school and health problems during treatment.

When I had to undergo emergency dialysis on exam day, I realized that nothing would ever be the same again, and my studies took a back seat. (P8)

Even when I studied, my mind was very scattered, and I couldn’t get my thoughts together. (P2)

## Being forced to change career choice

Many participants emphasized that they had dreamed of certain careers during childhood and adolescence, but these goals did not materialize due to their illness.

Before dialysis, I wanted to be a civil police officer… but we couldn’t study. (P11)

Some participants stated that they had turned to fields completely different from their dreams.

At that time, I wanted to be a teacher. We took breaks from classes … then I became an electrician. (P16)

## Discussion

### Psychological effects

It has been found that children undergoing PD and their mothers experience intense fear and shock at the time of diagnosis and treatment initiation, which is mostly related to lack of information and uncertainty [16]. In a qualitative study, children described the diagnostic process as “an unexpected and uncontrollable devastation,” and it was shown that their levels of fear increased when they were not adequately informed during this period [17]. The shock experienced during the diagnosis and initiation of treatment is a traumatic turning point for both children and parents. The sudden or unexpected diagnosis of kidney failure in children may make it difficult for them to accept that they are faced with a chronic disease requiring continuous treatment such as dialysis [18]. This study found that the sadness experienced by mothers during the treatment process and their feelings of pity for their children caused the children to feel more upset. It reveals that increased stress and emotional burden in families can negatively affect children’s emotional responses, particularly highlighting feelings of hopelessness and helplessness in the absence of social support [18]. It has been noted that overly protective parenting attitudes can limit feelings of independence during adolescence and may increase conflicts [7]. Additionally, peritoneal catheters, dialysis machines, and treatment-related visibility factors have been found to increase feelings of embarrassment and social shyness in children. Watson [19] states that body image is critical for social adjustment during adolescence, and that visible health conditions negatively affect self-confidence. The findings of this study indicate that participants experienced intense fear, shock, and sadness during the PD process in childhood. In particular, experiences related to the dialysis machine, catheter procedures, and the treatment environment were prominently reflected in participants’ statements. For example, one participant stated, “The sound of the machine frightened me a lot; I felt like my heart was going to jump out of my chest” (P12), clearly demonstrating the strong emotional impact of the treatment process on children. While these findings are consistent with the themes of fear and uncertainty reported in the literature [9, 10, 16], the present study also shows that participants re-evaluated these experiences in adulthood and interpreted them not only as negative experiences but also as a process contributing to the development of coping skills and resilience. This suggests that the experience of pediatric PD is not limited to short-term effects specific to the treatment period, but rather represents a multidimensional experience that may influence an individual’s lifelong psychosocial development.

### Daily life effects

Peritoneal dialysis treatment brings with it certain dietary restrictions. Changes in eating habits and strict dietary restrictions can affect children’s growth and development processes and make it difficult for them to adapt to eating patterns in social settings [20]. Restriction of dietary phosphorus may reduce the consumption of protein-containing foods in children undergoing dialysis; this may lead to protein–energy malnutrition and indirectly negatively affect growth and development [6]. It has been reported that protein–calorie malnutrition is common in children undergoing PD, with metabolic acidosis, chronic inflammation, and inadequate energy–protein intake among the causes of this condition [20]. The presence of a catheter in the abdominal area during PD, abdominal swelling associated with dialysis fluid, and visible changes in the skin directly affect children’s body image. Some studies have shown that these physical changes in dialysis patients can weaken self-esteem, lead to distortions in body image perception, and cause social withdrawal [21, 22]. PD treatment requires the use of multiple medications, and in pediatric patients, this complicates treatment compliance. It has been emphasized that difficulties in medication use increase, especially in cases where family support is insufficient, and that this situation can negatively affect treatment success [5]. The importance of the medications that children need to take during treatment should be discussed with both the children and their families, and the effects of the medications on the body should be explained.

This study revealed that repeated hospitalizations in children undergoing PD treatment were most commonly due to infection and high phosphorus levels. Peritonitis attacks are more common, especially during childhood, due to the immature immune system, and recurrent infections increase hospitalization rates and treatment costs. These findings highlight the necessity of meticulously implementing infection control protocols in pediatric patients. Infections occurring during treatment significantly limit both daily living activities and social and academic functioning [6]. Recurrent episodes of peritonitis, in particular, can trigger feelings of anxiety and hopelessness in patients by negatively affecting not only physical health but also psychological well-being and social participation [23]. Hyperphosphatemia is a common metabolic complication in children undergoing PD that may lead to hospitalization. Hyperphosphatemia is associated with bone-mineral imbalances and increased cardiovascular risks [24]; regular laboratory monitoring and dietary control are crucial as it may lead to permanent cardiovascular damage in later years [5].

Children undergoing PD develop various skills to cope with the stress caused by the treatment process in physical, psychological, and social terms. These skills are crucial for maintaining emotional balance, sustaining social adaptation, and improving overall quality of life. Digital interactions such as social media and watching movies help children stay connected to their social environment, while skills such as imagination help children keep their hopes alive and maintain emotional motivation [25]. It has been noted that patients on hemodialysis and PD use personal care and esthetic-focused activities (such as doing makeup and caring for their personal appearance) as a coping method that increases self-esteem and reduces the feeling of loss of control associated with the disease [26]. In this study, children’s writing about their emotions emerges as a skill they use to cope with stress. Expressing emotions is known to function as a healthy coping mechanism. Environments that allow children to express their emotions are critical in reducing emotional burden and facilitating the coping process [13]. It has been observed that some children undergoing PD turn to emotional eating to cope with the chronic stress and emotional distress they experience [7]. It has been noted that acceptance and active adaptation in children are associated with psychosocial processes such as participation in treatment, development of self-regulation skills, positive reappraisal, and future-oriented thinking. Such active participation during PD enables children to demonstrate stronger adaptation to the treatment process.

### Social effects

Social isolation is one of the most commonly reported psychosocial difficulties among children and adolescents undergoing CKD and PD. It has been determined that dialysis treatments administered at home lead to withdrawal from peer relationships and an increase in feelings of loneliness [27]. Another study also emphasizes that life restrictions associated with the dialysis process limit children’s communication with their social environment, reduce their participation in school and social activities, and negatively affect their psychological well-being [6]. PD treatment limits children’s participation in vacations, sports activities, and social outings. In particular, the regular procedures required by the treatment process, dependence on machines and materials, and the risk of infection make activities such as swimming, camping, or long-term travel impossible, leading to negative effects on social development and the sense of belonging [2]. Qualitative studies also indicate that reduced participation in social activities increases feelings of loneliness, social withdrawal, and exclusion; it can negatively affect the development of social skills [11]. It is stated that the emotional burden experienced by families in PD care increases social withdrawal and introversion tendencies in children [28]. The PD process has been reported to decrease children’s participation in social activities, weaken peer relationships, and increase withdrawal behaviors [13]. It has been emphasized that children living with chronic illness experience shyness, social isolation, and decreased participation in social activities in their peer interactions [29]. PD rooms set up in home environments or hospitals play a significant role in the lives of children. However, these environments cannot always meet children’s social and emotional needs due to factors such as hygiene rules, limited physical space, a quiet atmosphere, and dependence on technical equipment [16]. It has been noted that the closed and routine-based physical environment of PD limits individuals’ social interactions and restricts their daily living activities [11]. Social support systems provided by family, peers, and health professionals have been shown to significantly improve children’s quality of life and psychological resilience. This support reduces feelings of loneliness while enabling children to reintegrate into their social environment and maintain emotional well-being [25]. Findings obtained within the scope of the SONG-PD project have revealed that sharing their experiences with others who have undergone similar processes reduces patients’ emotional burden, improves their coping skills, and positively affects their quality of life [30].

### Academic effects

Treatment-related physical limitations, frequent hospital visits, psychological stress, and fatigue can cause various difficulties in education [6]. CKD in childhood, and especially processes requiring PD, causes school absenteeism. The annual rate of ≥ 18 days of school absenteeism among children with CKD is 17.3%, while in the general population, this rate is only around 2.7%, indicating that absenteeism is approximately 6 times higher in the CKD group [31, 32]. Additionally, it has been noted that among the most common causes of chronic absenteeism in children are factors such as doctor appointments, feeling unwell, and being marginalized at school [33]. Due to frequent hospital visits, procedures, fatigue, and fluctuations in health status, some children are forced to take extended breaks from their education. Taking breaks from education and absenteeism in children with long-term CKD can lead to falling behind in classes and low motivation [33, 34]. Fatigue, anemia, metabolic imbalances, and developmental delays in children with CKD can lead to decreased performance, particularly in subjects requiring cognitive intensity such as mathematics and language [6]. Children with CKD have been shown to experience academic disadvantages due to inattention, slower learning speed, and difficulties participating in class [33]. In pediatric patients, fatigue is reported to be accompanied not only by physical exhaustion but also by significant impairments in emotional and cognitive functioning, which can negatively affect attention, motivation, and school performance [35]. It has been reported that individuals living with CKD during childhood tend to gravitate toward jobs in adulthood that require less physical exertion, offer flexible hours, and can accommodate their health status [6]. In young adulthood, it was found that unemployment rates and disability benefit receipt rates were significantly higher among individuals with a history of CKD compared to healthy peers [34].

### Strengths and limitations of the study

This study has some limitations. First, the use of the snowball sampling method may have led to sampling bias, as participants may generally consist of individuals with similar experiences or social networks. In addition, the inclusion of only individuals who completed PD treatment during childhood and reached adulthood may have caused survivorship bias. This may have led to the inability to reflect the experiences of individuals with a more severe clinical course or those who were lost during the treatment process and may have resulted in the findings being specific to a particular patient group. The other limitation of the study is that the findings obtained are specific to a particular group of participants and cannot be generalized to the entire population. The fact that the research data is based on individuals’ personal accounts requires the memory factor, especially in terms of the need for retrospective recollections. The retrospective design of the study also carries a risk of recall bias; participants may have reinterpreted their past experiences over time or may have had difficulty recalling certain details. This may affect the accuracy and comprehensiveness of the data.

One of the strengths of this study is that adults who received PD during childhood were able to report their experiences related to the entire treatment process, including diagnosis, initiation of treatment, home care, possible complications, and the reorganization of life after treatment. This allows participants to evaluate the treatment not only in terms of immediate experiences but also in a holistic manner, considering its long-term effects. This feature distinguishes the study from those conducted with children who are currently undergoing PD.

## Conclusion

In conclusion, the study allowed adults who received PD treatment during childhood to observe the impact of their treatment on their lives and to explain the most challenging and life-affecting aspects of managing the disease during their childhood. PD in childhood causes limitations in children’s physical and psychological well-being, as well as in their social and academic lives. PD treatment process should include not only monitoring physical indicators but also continuous tracking of psychosocial changes and academic progress. The findings of this study reveal several important nuances. While the literature mostly emphasizes immediate experiences specific to the treatment environment, participants in this study also expressed the long-term effects of these experiences; in particular, their reflections on personal life, adaptation, career choice, and social relationships became more evident. During childhood, the family’s—particularly the mother’s—adaptation to treatment and support for the child’s adjustment and coping skills will help ensure that the child grows up to be a healthy adult and will reduce any regrets regarding the treatment process during childhood. In particular, the ability of mothers and family members to adapt to PD treatment and manage their own emotions affects how the child perceives and manages the treatment. The findings of this study indicate that focusing solely on medical indicators in the care of children undergoing PD is not sufficient, and that early experiences may lead to long-term psychosocial effects. Therefore, it is important that care approaches for children and their families during the PD treatment process are planned in a more structured and individualized manner. In particular, providing children with age-appropriate and understandable information from the time of diagnosis may help reduce uncertainty and fear related to the treatment process. In addition, children should be gradually and supportively prepared for the treatment environment (e.g., dialysis machines, catheter procedures, and care practices) in order to reduce related fears. Furthermore, it is important to recognize early the impact of the treatment process on social and educational life and to plan interventions that support children in maintaining peer relationships. The findings in our study suggest that such support may influence not only short-term adaptation but also long-term psychosocial development. Therefore, it is recommended that psychosocial assessments be incorporated into routine care practices and that children at risk be identified early and provided with appropriate support. Furthermore, in cases where children are unable to attend school due to PD, providing them with remote access to live online classes can be effective in supporting their social development with their peers and improving their academic performance. This situation will contribute to children completing education and acquiring professions in line with their abilities. According to these research findings, a multidisciplinary team consisting of a nephrologist, peritoneal dialysis nurse, psychologist, child development specialist, nutritionist, social worker, and school counselor is needed, along with a child-centered treatment and follow-up process supported by family and peers. Peritoneal dialysis nurses play a key role in managing the process, collaborating with specialists on the team. Future research should examine similar patient groups in different sociocultural contexts to assess the generalizability of these findings. Furthermore, studies using longitudinal designs are recommended to more comprehensively reveal the lifelong effects of treatment experiences during childhood. Additionally, research evaluating the impact of early psychosocial support and educational interventions on long-term outcomes is expected to make significant contributions to the literature.

## Supplementary Information

Below is the link to the electronic supplementary material.

Graphical abstract (PPTX 74.8 KB)

## Data Availability

The data that support the findings of this study are available from the corresponding author upon reasonable request. Due to the qualitative nature of the data and to protect participant confidentiality, transcripts cannot be shared publicly.
